# Critical transitions and evolutionary hysteresis in movement: Habitat fragmentation can cause abrupt shifts in dispersal that are difficult to revert

**DOI:** 10.1002/ece3.10147

**Published:** 2023-05-29

**Authors:** Monique de Jager, Merel Soons

**Affiliations:** ^1^ Quantitative Biodiversity Dynamics, Department of Biology Utrecht University Utrecht The Netherlands; ^2^ Ecology & Biodiversity Group, Department of Biology Utrecht University Utrecht The Netherlands; ^3^ Animal Ecology Group Netherlands Institute of Ecology (NIOO‐KNAW) Wageningen The Netherlands; ^4^ Copernicus Institute of Sustainable Development, Utrecht University Utrecht The Netherlands

**Keywords:** eco‐evolutionary dynamics, local dispersal, long‐distance dispersal, movement strategies, restoration, seed dispersal

## Abstract

Under habitat fragmentation, plant species' survival hinges on the ability of individuals to disperse from one habitat patch to another. While there is evidence that severe habitat fragmentation leads to evolution of reduced dispersal ability and that such decreased mobility is generally detrimental for species' survival, it is unknown whether species adapt via a gradual loss in dispersal ability or via a sudden shift from frequent to infrequent dispersal between patches (i.e., a critical transition). Using both a spatially explicit deterministic and individual‐based stochastic model of hydrochorous seed dispersal, we show that a small increase in inter‐patch distance can generate an abrupt shift in plant seed dispersal strategy from long to short distances. Most importantly, we found that a substantial increase in connectivity between habitat fragments is required to reverse this loss of long‐distance dispersal, due to an evolutionary hysteresis effect. Our theory prompts for re‐consideration of the eco‐evolutionary consequences of habitat fragmentation as restoring habitat connectivity may require restoration of much higher connectivity levels than currently assumed.

## Graphical abstract

We suggest that restoring habitat connectivity may require restoration toward much higher connectivity levels than currently assumed. Using a spatially explicit, individual‐based modeling approach, we show that a small increase in inter‐patch distance can generate an abrupt shift in plant seed dispersal strategy from long to short distances. Most importantly, we found that a substantial increase in connectivity between habitat fragments is required to reverse this loss of long‐distance dispersal, due to an evolutionary hysteresis effect.

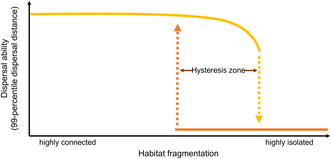

## 1 INTRODUCTION

Globally, habitat fragmentation is a major cause of population declines and biodiversity loss (Diffenbaugh & Field, 2013; IPBES, 2019). Currently, 37% of grasslands, 70% of forests, and 60% of the largest rivers are fragmented (Haddad et al., 2015; Keeler‐Wolf, 2001; Revenga et al., 2000), with remaining natural habitat patches becoming smaller, fewer, and farther apart, resulting in unprecedented rates of species extinction (IPBES, 2019; Tilman et al., 2017). In this increasingly fragmented world, nature conservation and restoration activities often concentrate on reinstating connections between patches by securing and restoring nearby suitable locations or implementing corridors. Yet, such efforts are often ineffective (Race & Fonseca, 1996; Suding, 2011). When habitat conditions are sufficiently restored, the lack of success in restoring species populations is often attributed to insufficient nearby source populations to initiate recolonization (Brederveld et al., 2011; Donath et al., 2003; Sundermann et al., 2011). However, when quantified, the availability of source populations in the surrounding landscape only explains the lack of recolonization to a limited extent (e.g., ca. 20% in restored streams; Brederveld et al., 2011) and other, unknown mechanisms are likely to also contribute substantially. It is of critical importance to identify these mechanisms, as failure of species to recolonize translates into failure to restore biodiversity and biodiversity‐dependent ecosystem services (Isbell et al., 2015).

Restoration activities are based on the assumption that species' dispersal abilities have been unaffected by the fragmented situation, leaving remaining populations well equipped to (re)colonize restored habitat patches (Honnay et al., 2002). Yet, dispersal is fundamental to an organisms fitness and is therefore under strong selection (Baguette et al., 2013). While habitat fragmentation can affect organisms in many ways, accumulating theoretical and empirical evidence points out that increased habitat fragmentation may lead to the evolution of short‐distance dispersal (Cheptou et al., 2008, 2017; Cody & Overton, 1996; Gandon & Michalakis, 1999; Heino & Hanski, 2001; Legrand et al., 2017; Lindenmayer & Fischer, 2013; Travis et al., 2010; Travis & Dytham, 1999). In highly fragmented conditions, (rapid) evolution of short‐distance dispersal strategies increases seed deposition within the parental patch while reducing the proportion of propagules lost to outside, uninhabitable areas. However, due to such a loss of dispersal capacity, colonization of new areas becomes impossible. Populations may thus become extremely vulnerable to local disturbances, gene flow between populations impaired, and networks of interactions with other species weakened (Bonte & Dahirel, 2017; Leibold et al., 2004; Ronce, 2007). In order to forecast this kind of eco‐evolutionary dynamics and mitigate its effects through management and planning (Tilman et al., 2017; Travis et al., 2010), crucial knowledge is required regarding how altering habitat fragmentation affects the evolution of dispersal strategies.

In plants, an increasing body of evidence suggests that populations in long‐term fragmented landscapes have indeed lost the ability to disperse over long distances (Cheptou et al., 2008, 2017; Legrand et al., 2017; Lindenmayer & Fischer, 2013; Travis et al., 2010). One reason for this may be the loss or modification of dispersal vectors in fragmented landscapes (Cordeiro & Howe, 2001; Ozinga et al., 2009). Another reason may be genetic loss of dispersal ability (Cheptou et al., 2008; Ronce, 2007), either resulting from inbreeding depression or genetic drift due to small population sizes (Cheptou & Donohue, 2011) or caused by evolutionary modification of seed dispersal traits in plant populations inhabiting fragmented landscapes (Cheptou et al., 2008, 2017).

**FIGURE 1:**
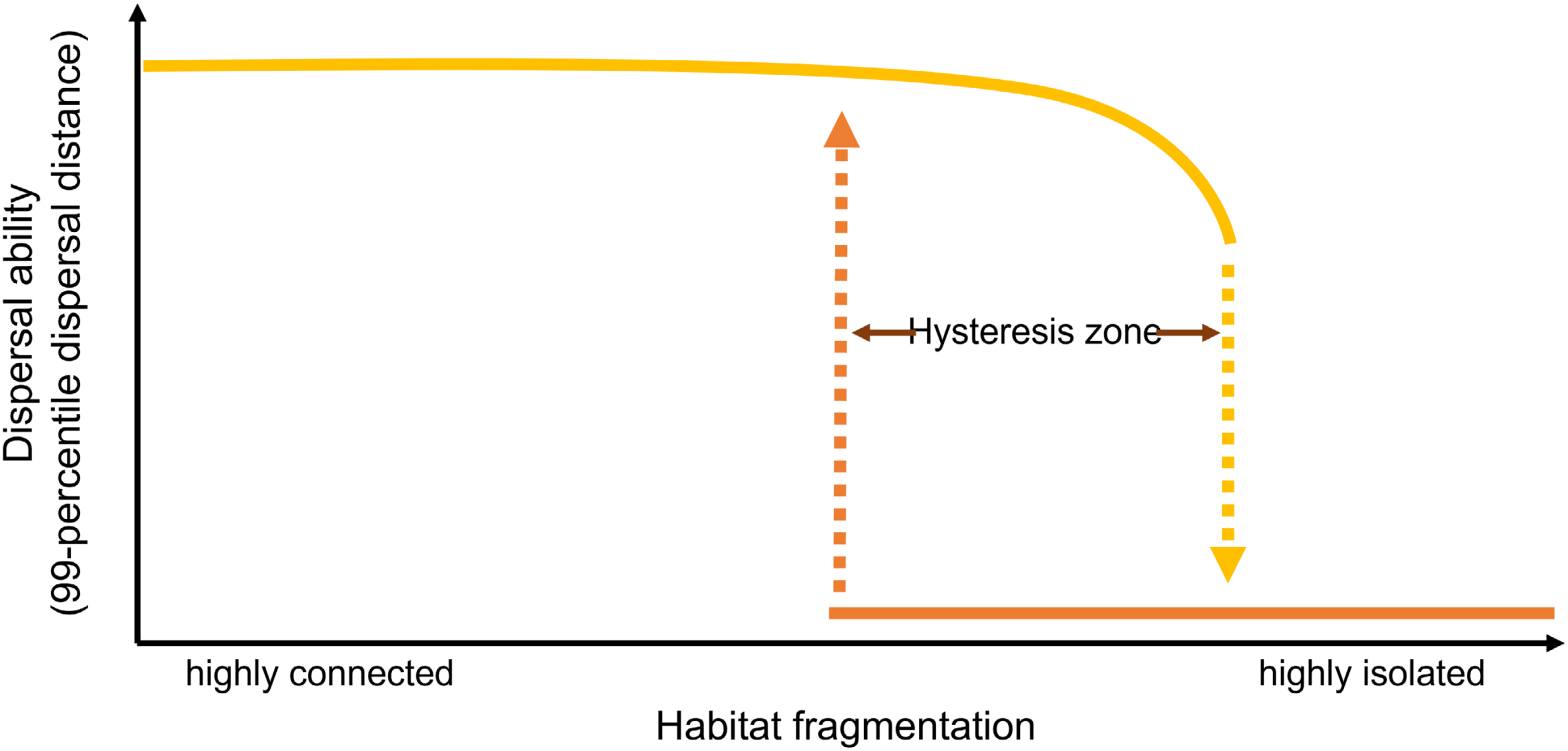
Hypothesized effect of habitat fragmentation on dispersal strategy, taking plant seed dispersal via water as an example. The yellow line indicates evolution of dispersal ability with increasing levels of habitat fragmentation; the orange line shows the evolution of dispersal ability with decreasing habitat fragmentation levels. In the hysteresis zone, the dispersal ability that evolves depends on the previous state of the system.

We theorize that if loss of dispersal has a genetic basis, ongoing habitat fragmentation and restoration can lead to a hysteresis effect in dispersal evolution (Figure 1). Hysteresis is a phenomenon that occurs when the state of a system depends on the history of past perturbations (Beisner et al., 2003). In our case, depending on past habitat fragmentation or defragmentation, alternative dispersal strategies can evolve in (currently) similar environments (Figure 1). Increasing fragmentation may result in a tipping point in dispersal capacity: once long‐distance dispersers can no longer colonize other habitat patches, those organisms that disperse over short distances and remain within the boundaries of the source population are more likely to survive, given the fact that far‐dispersed propagules are lost to uninhabitable areas (Balkau & Feldman, 1973; Gandon & Michalakis, 1999; Hastings, 1983; Holt, 1985). Within a community, this process leads to short‐distance dispersing species outcompeting long‐distance dispersing species (Liao, Li, Hiebeler, El‐Bana, et al., 2013; Liao, Li, Hiebeler, Iwasa, et al., 2013), and within a population, mutations resulting in short‐distance dispersal suddenly become advantageous (Cheptou et al., 2008). We hypothesize that a long‐distance dispersal strategy can abruptly evolve into short‐distance (within‐patch dispersal), once habitat patches become unreachable. Once local dispersal has evolved, we hypothesize that a substantial decrease in the distance between habitable areas is required to reverse the loss of dispersal capacity (a hysteresis effect; Figure 1), as gradual changes in dispersal capacity may not evolve when habitable patches remain out of reach.

Collecting empirical evidence establishing the hypothesized evolutionary trajectories would involve experimental habitat manipulation over many generations, which would require an extensive period of time that is not available given the urgency of mitigating effects of habitat fragmentation. Therefore, we use a simple model, based on a well‐documented and data‐rich system: dispersal by water of shoreline plant species. Dispersal of shoreline plant seeds by stream or river water flow provides a well‐studied, mostly unidirectional and linear study system: shoreline plants produce seeds that are released onto, or taken up by, stream or river water, which transports them downstream until they are deposited on the bank (de Jager et al., 2019; Merritt & Wohl, 2002; Nilsson et al., 2010; Soons et al., 2017). This type of dispersal is the predominant dispersal mode for shoreline plants and regulates population survival as well as spatial configuration (Nilsson et al., 2010). Recent advances established (i) that shoreline plants have long‐floating seeds adapted to dispersal by surface water flows as primary dispersal mechanism, securing eventual deposition at the shoreline (Soons et al., 2017), and (ii) that larger seeds are dispersed over longer distances by river water flow, following a simple exponential relation between seed size and dispersal distance (de Jager et al., 2019), while (iii) upstream dispersal occurs via other, usually accidental, vectors to which plant species do not have clear adaptations (Wubs et al., 2016). Riparian plant habitat is fragmented (Lake, 2000) and ongoing habitat degradation and restoration both further affect this heterogeneity (Lake et al., 2007). We used this well‐known simple system to provide a realistic proof‐of‐principle of our hypothesized hysteresis effect in dispersal evolution as a consequence of habitat fragmentation and restoration events.

## 2 METHODS

**FIGURE 2:**
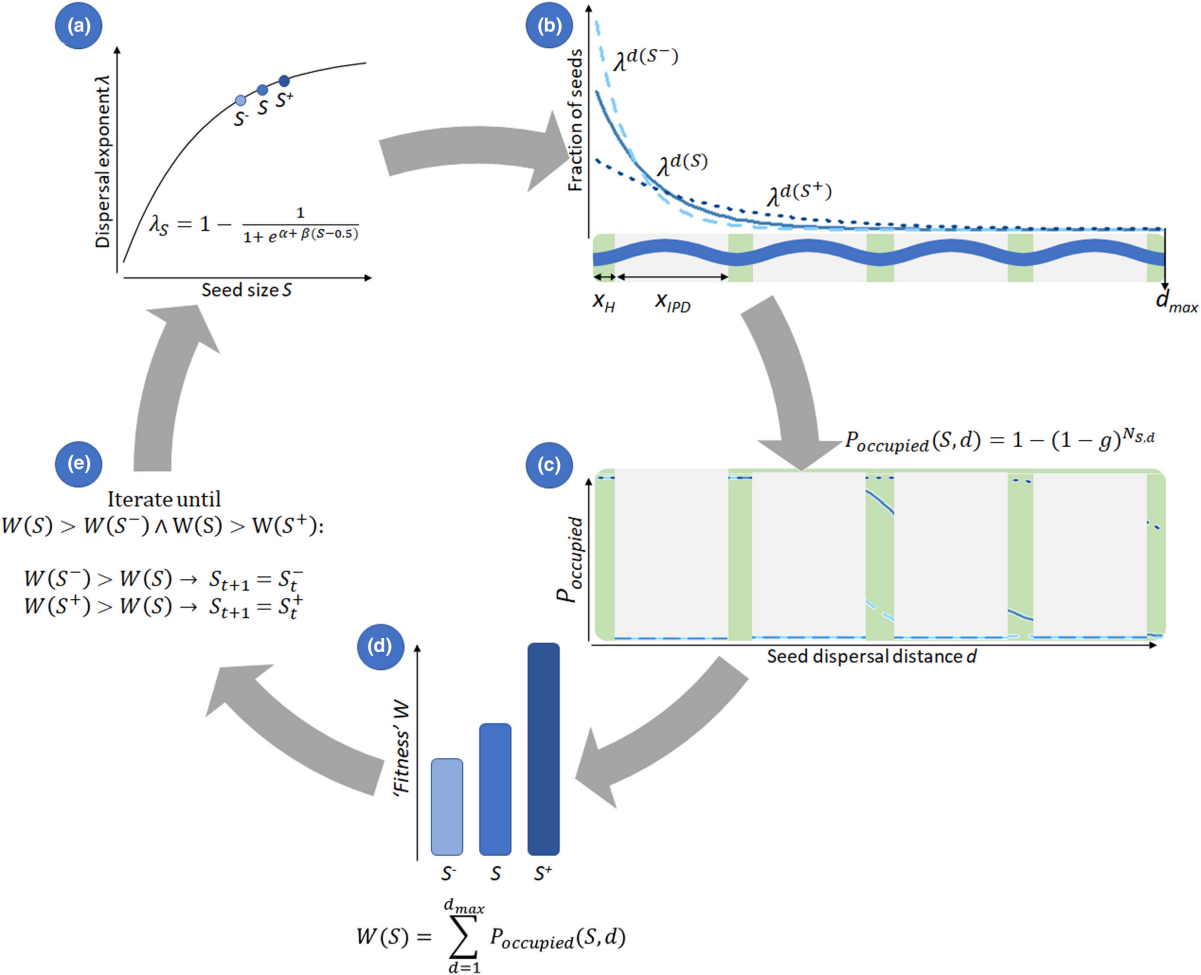
Simplified overview of the deterministic model, where we seek the seed size (*S*) that results in the most optimal dispersal kernel, given the environment and assuming small mutations in seed size *S* (*S* ^−^ and *S* ^+^). (a) Seed size *S* determines the dispersal exponent *λ*. (b) The fraction of seeds that falls onto each habitable cell (in habitable patches of size *X*_*H*_) depends on *λ*. (c) Per habitable cell, the probability that it becomes occupied (at least one seed germinated and survived) depends on the number of seeds dispersed to that cell. (d) Fitness per seed size *S*, *S* ^−^ and *S* ^+^ are calculated. (e) If *S* ^−^ or *S* ^+^ has a larger fitness than *S*, *S* will hold the value of the more optimal seed size in the next iteration.

To provide a proof‐of‐principle of critical transitions and hysteresis in dispersal evolution, we simulated dispersal of shoreline plant seeds by water and assessed the potential for evolution of dispersal strategies in fragmented landscapes. For this purpose, we created a simple, computationally fast, deterministic model, and a more realistic, stochastic, individual‐based model, to cover a range from simplicity to complexity that allows detection of relevant underlying mechanisms. As dispersal of shoreline plants by rivers and streams is mostly unidirectional, the models are spatially explicit in one dimension. In both models, we used seed size as the mutable trait under selection and the colonized habitat area (expressed in average number of grid cells colonized by a plant's seeds) as the measure of fitness to be maximized. In the deterministic model, we simulated seed dispersal by one individual in an otherwise empty environment, whereas in the stochastic model, we simulated seed dispersal by multiple individuals. A list of parameters, their definitions, and values are provided in Table 1. A simplified overview of the deterministic model is shown in Figure 2.

**TABLE 1:** List of parameters, their definitions, and values.

Parameter	Definition	Value
*α*	Parameter defining the center of the sigmoidal relation between seed size (*S*) and the decay exponent (*λ*)	3.3–6.1
*β*	Parameter defining the slope and center of the sigmoidal relation between seed size (*S*) and the decay exponent (*λ*)	−0.1 to 0.2
*λ*_*S*_	Exponent of exponential decay function of dispersal distance (function of seed size *S*)	0–1
*μ*	Mutation rate	0.001^b^
*c*	Production cost parameter	0.00001–1.5
*c*_1_	Cost per seed volume	0.0001
*d*	Dispersal distance (in grid cells)	1 – *X*_max_
*d*_0.5_	Median dispersal distance of the smallest seeds (*S* = 0.5 mm)	20–300
*d*_30_	Median dispersal distance of the largest seeds (*S* = 30 mm)	50–750
*d*_max_	Maximum dispersal distance (boundary of the simulated environment, in cells)	50,000
*g*	Probability of germination and survival	0–1
*N*_gens_	Number of generations	10,000^b^
*N*_*j*_	Number of seeds dispersed by plant *j* (function of seed size and production cost)	0–1000
*N*_tot_	Total number of seeds to disperse	10,000^a^; 1000^b^
*N*_*x*_	Number of seeds dispersed to a grid cell at distance *x* from the parental cell.	0 – *N*_tot_
*P*_*x*_^*+*^	Probability that a grid cell at distance *x* from the parental cell will be occupied by the next generation	0–1
*S*	Seed size (in mm)	0.5–30
*X*_*H*_	Number of grid cells per habitat patch	50
*X*_IPD_	Number of uninhabitable grid cells between subsequent habitat patches	0 – *X*_max_
*X*_max_	Maximum inter‐patch distance, in grid cells	5000^a^, 1000^b^

^a^ Deterministic model parameter value.

^b^ Stochastic model parameter value.

### 2.1 Landscape

We simulated semelparous seed dispersal in a unidirectional, linear system consisting of habitat patches (each consisting of 50 grid cells per patch (*X*_*H*_ = 50)) that are separated by inter‐patch distances (IPDs) containing non‐habitable patches (each consisting of *X*_IPD_ cells per patch). A grid cell can contain only one plant individual. Between habitat patches, we first increased inter‐patch distances (*X*_IPD_) from 0 to *X*_max_ grid cells between model runs and subsequently decreased the inter‐patch distance again from *X*_max_ grid cells back to 0, while using the seed sizes that evolved in the previous run as the initial seed sizes in the next run to examine hysteresis effects.

### 2.2 Seed dispersal

Seed dispersal distance of shoreline plants depends on seed size (de Jager et al., 2019). Seed size was defined by seed length, which varies for shoreline plants (helophytes) between 0.5 and 48 mm, with 95% of the seed lengths between 0.5 and 27 mm (de Jager et al., 2019; Kleyer et al., 2008). In our models, we therefore used a range of seed sizes between 0.5 and 30 mm, with plants producing only a single seed size. The number of seeds dispersed depends on the seed size, as larger seeds are costlier to produce (see below).

Seed dispersal by water depends on the speed of the water flow, the floating time of the seeds and the probability that a seed becomes entrapped on a river or stream bank, which ends the dispersal event. For seeds with very long‐floating times (as is common for shoreline plants; Soons et al., 2017), probability of entrapment depends on seed size (de Jager et al., 2019). A field study on seed dispersal by water showed that experimental seed dispersal distance data are best represented by an exponential dispersal kernel for which the decay parameter depends on seed size following a sigmoid relation (de Jager et al., 2019). Assuming no differences in entrapment probability between habitat patches and uninhabitable areas, the fraction of seeds remaining in the water column after dispersing a distance *d* (in cells) thereby follows an exponential decay function:

(1) $$F_{d}=\lambda^{d},$$

where *λ* is the decay exponent (de Jager et al., 2019). As *λ* cannot be smaller than 0 or larger than 1 (in which case the frequency would increase rather than decrease with distance), we assume that *λ* is a sigmoidal function of seed size (*S*):

(2) $$\lambda_{S}=1-\frac{1}{1+e^{\alpha +\beta \left(S-0.5\right)}}$$

where *α* and *β* are the model parameters that define the slope and shape of the sigmoid curve (de Jager et al., 2019). The 0.5 in equation 2 represents the minimum seed size (0.5 mm), for which *β(S‐0.5)* = 0. As *α* and *β* are not ecologically meaningful and therefore not easily interpreted, we hereafter parameterized the dispersal kernel using the median dispersal distances (*F*_*d*_ = 0.5) of the smallest and largest seeds considered in our model (0.5 and 30 mm, respectively): *d*_0.5_ and *d*_30_ at *F*_*d*_ = 0.5. The relations between *d*_0.5_ and *d*_30_ at *F*_*d*_ = 0.5 and *α* and *β* are described in Appendix 1.

### 2.3 Seed size evolution

Both models depend on small mutations in seed size; if seed size does not mutate gradually, hysteresis effects are not expected to occur. In the deterministic model, seed size evolved toward the size S* whose relative fitness is higher than that of somewhat larger or smaller seeds (*S* ^*−*^ = *S* – 0.01 mm and *S* ^*+*^ *= S +* 0.01 mm) and can be attained from the initial seed size (the seed size S* in the previous run with a smaller or larger inter‐patch distance, modeling increasing or decreasing habitat fragmentation, respectively). Relative fitness was determined by combining a plant's colonization successes at all habitat cells. In the stochastic model, seed size evolution depended on the mutation rate (*μ*) and the distribution of seeds after dispersal across habitable cells. In this model, we did not select the seed size that resulted in the highest relative fitness directly; seed size evolution was governed by the stochastic processes of dispersal and consequent germination and survival of a random seed per habitat cell.

### 2.4 Seed size evolution: deterministic model

In the deterministic model, we calculated, per habitat cell, the probability that at least one of the seeds dispersed to this cell germinated and survived. For a given seed size *S*, the number of seeds dispersed to a grid cell at distance *d* from the parental cell is given by:

(3) $$N_{d}=\frac{N_{\mathrm{tot}}}{\lambda^{d_{\max}}-\lambda^{0}}∙\left(\lambda^{d+1}-\lambda^{d}\right)∙\left(1-c\right).$$

Here, *N*_tot_ is the total number of seeds to disperse, *d*_max_ and 0 are the edges of the simulated environment (*d*_max_ = 5000 cells), and *c* is the production cost parameter:

(4) $$c=\frac{c_{1}}{6}∙\pi ∙S^{3},$$

where the cost *c* is considered to be linearly proportional to the seed volume, with calibrating constant *c*_1_ (*c*_1_ = 0.0001). We assume (i) round seeds (ii) that large seeds are more costly to produce, and (iii) that large seeds have a higher chance of survival. This trade‐off between production cost and survival chance is incorporated in *c* to simplify the model. Naturally, the shape of the seed size cost function will affect results. In our sensitivity analysis, we vary the value of *c*_1_ to examine its effect.

In the deterministic model, we did not include any form of competition, as only one plant individual was simulated per run. The only competition that was implicitly included was between kin: of all the seeds that a plant dispersed to the same grid cell, only one could survive. Given a certain probability of germination and survival, *g*, the probability that a grid cell will be occupied (*P* ^*+*^) depends on the number of seeds dispersed to it:

(5) $$P_{d}^{+}=1-{\left(1-g\right)}^{N_{d}},$$

where *N*_*d*_ is the number of seeds dispersed to the grid cell at distance *d* from the parental grid cell. In the current model, we set *g* to 0.3 for habitat patches and 0 for uninhabitable patches. In our sensitivity analysis, we varied the value of *g* to examine its effects. We used the sum of $P_{d}^{+}$ between 0 ≤ *d* ≤ *d*_max_ grid cells as a measure of fitness, as it indicates the average number of grid cells and thus total habitat area that may be colonized by a plant's seeds. We compared these fitness measures between plants that produce seeds of a certain size *S* (0.5 < *S* ≤ 30 mm) to fitness of plants that produce slightly larger or smaller seeds (*S* ^*−*^ *= S* – 0.01 mm and *S* ^*+*^ *= S +* 0.01 mm). When fitness of *S* ^−^ or *S* ^+^ is higher than that of S, seed size evolved and new comparisons were made between the new fitness values, until seed size *S* ^*^ was reached whose fitness was higher than that of both *S* ^*−*^ and *S* ^*+*^. With this model, we thus disregard the time it takes for seed size *S* ^*^ to evolve, the probability that multiple seed sizes may evolve simultaneously and stochasticity in fitness. With the seed size evolved in an environment with inter‐patch distance *X*_IPD_ (starting with *X*_0_ = 0), we initiated seed size evolution in the environment with inter‐patch distance *X*_IPD+1_, until *X*_max_ (*X*_max_ = 5000) was reached, after which we decreased inter‐patch distance again to 0, one grid cell at a time. All calculations were run once; since our model is deterministic, there is no need for replications of runs.

### 2.5 Seed size evolution: stochastic model

In the stochastic model, boundaries are continuous. Initially, simulations start with *X*_IPD_ = 5 m and all habitable cells hold a plant that produces seeds with intermediate seed size *S* = 20 mm. Plants one by one (in a random order) disperse their seeds, until either all seeds are dispersed or all habitable cells are occupied by the next generation of seedlings. The number of seeds dispersed by plant *j* depends on seed size and the parameter *N*_tot_:

(6) $$N_{j}=N_{\mathrm{tot}}∙\left(1–c\right),$$

where *c* is calculated as in equation 4. Dispersal distance of the randomly selected seed is drawn from an exponential distribution (equation 1). If the seed disperses to a habitable cell that is unoccupied by a seedling (ignoring the parent generation), it can germinate and survive there with probability *g*. Seeds dispersed to inter‐patch locations or to cells already occupied by other seedlings have zero survival and germination probability. Established seedlings can have a larger or smaller seed size (+ or – 1 mm) than their parent with a mutation rate *μ* = 0.001. After a fixed number of generations (*N*_gens_), we recorded the population's seed size distribution and increased the inter‐patch distance by 5 m (i.e., 5 cells). When *X*_IPD_ = *X*_max_ = 1000 m was reached, we again decreased the inter‐patch distance with 5 m per *N*_gens_ generations. This model thus simulates evolution of dispersal distances and includes stochasticity and competition, but is computationally much more demanding, which is why it was run for a more limited set of conditions.

### 2.6 Sensitivity analysis

To evaluate how model results depend on parameter values, we ran additional model runs varying the parameters patch size (10, 50, 100, and 500 grid cells), survival and gemination (*g* = 0.9, 0.6, 0.1, and 0.01), seed size cost (*c*_*i*_ = 0.000001, 0.00001, 0.001, and 0.01), total number of seeds to disperse (*N*_tot_ = 100, 1000, and 100,000), number of generations per inter‐patch distance (*N*_gens_ = 10, 100, 1000, and 10,000), and mutation rate (*μ* = 0.01, 0.001, 0.0001).

## 3 RESULTS

### 3.1 Critical transitions and hysteresis in dispersal evolution

**FIGURE 3:**
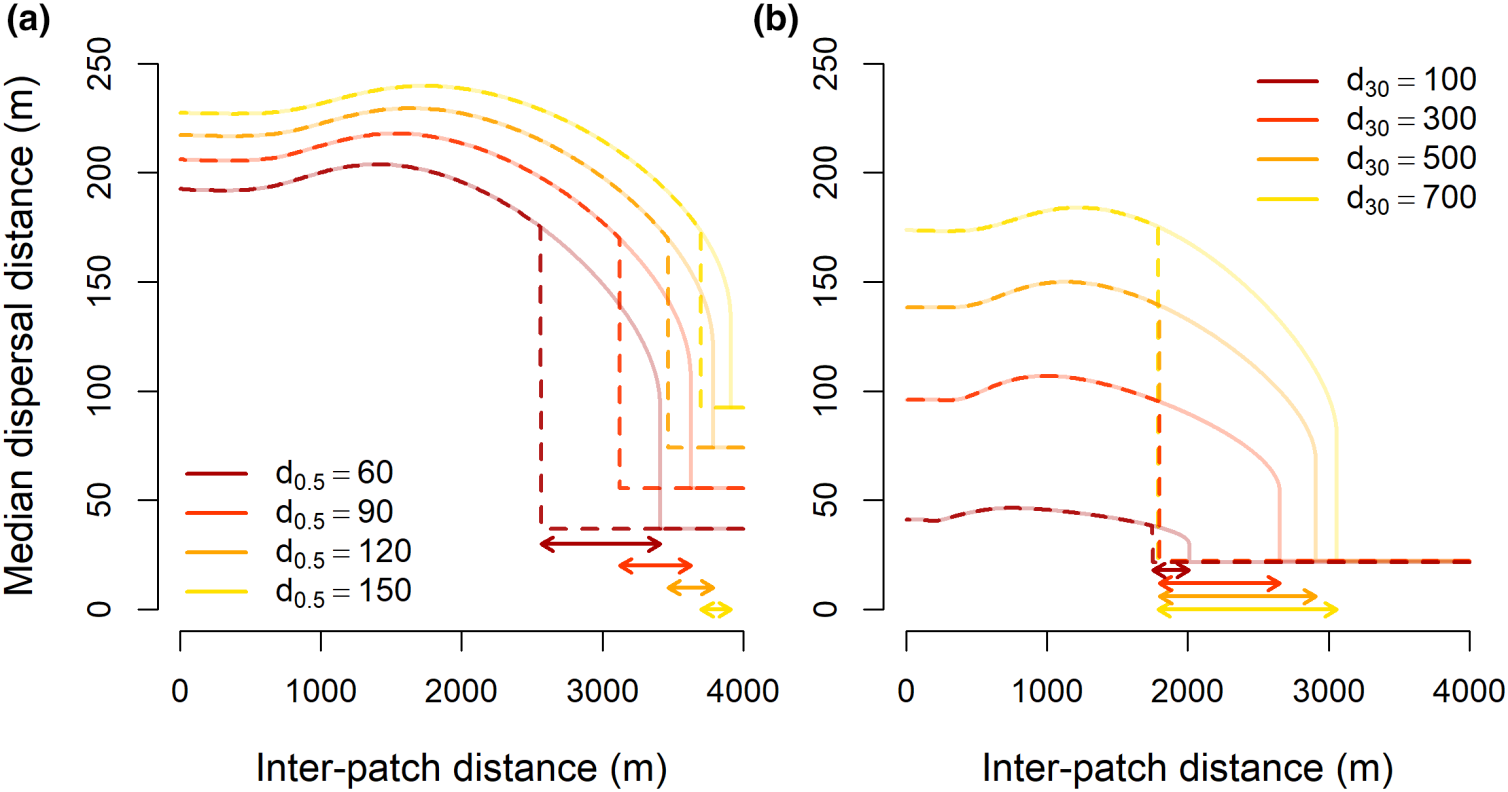
Examples of median dispersal distances that evolved in the deterministic model. First, inter‐patch distances were increased (solid lines); subsequently, we decreased inter‐patch distances (dashed lines). In the area between the dashed and solid vertical lines a hysteresis zone occurs, where evolved dispersal capacity depends on fragmentation history. In (a), we show evolved median dispersal distances when the median dispersal distance of the smallest seeds (*d*_0.5_) is 60, 90, 120, or 150 m (illustrated by the different colors) and the median dispersal distance of the largest seeds (*d*_30_) is 700 m. In (b), the median dispersal distance of the smallest seeds (*d*_0.5_) is 30 m, and the median dispersal distance of the largest seeds (*d*_30_) is 100, 300, 500, or 700 m (illustrated by the different colors). Other parameter values were kept constant at patch size *X*_*H*_ = 50, total number of seeds dispersed per plant *N*_tot_ = 10,000, survival and germination rate *g* = 0.3, and seed production cost *c*_1_ = 0.0001. Arrows indicate the hysteresis zones.

**FIGURE 4:**
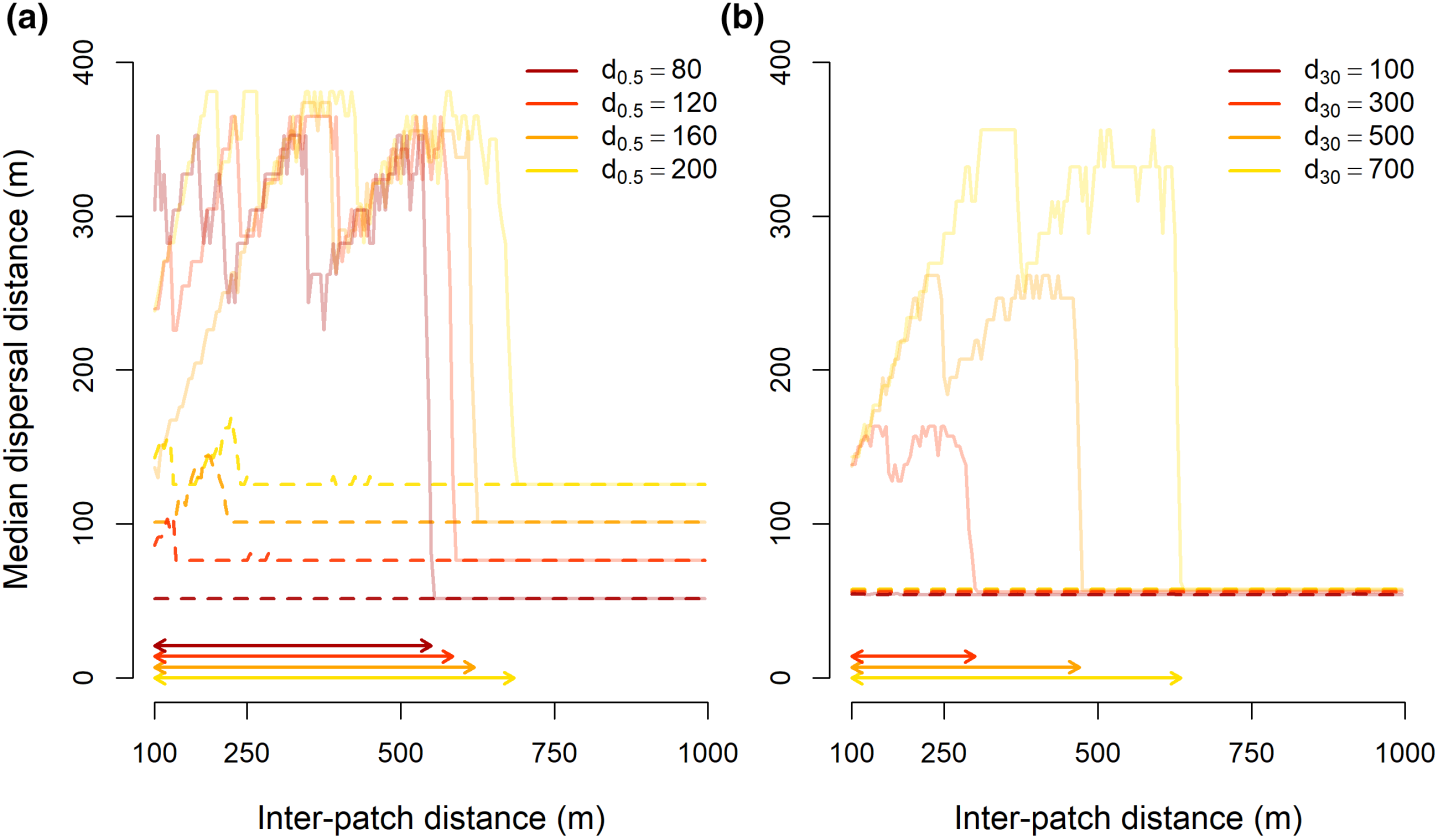
Examples of median dispersal distances that evolved in simulations with the stochastic model. First, inter‐patch distances were increased (solid lines); subsequently, we decreased inter‐patch distances (dashed lines). In the area between the dashed and solid vertical lines a hysteresis zone occurs, where evolved dispersal capacity depends on fragmentation history. In (a), we show evolved median dispersal distances when the median dispersal distance of the smallest seeds (*d*_0.5_) is 80, 120, 140, or 200 m (illustrated by the different colors), and the median dispersal distance of the largest seeds (*d*_30_) is 700 m. In (b), the median dispersal distance of the smallest seeds (*d*_0.5_) is 90 m and the median dispersal distance of the largest seeds (*d*_30_) is 100, 300, 500, or 700 m (illustrated by the different colors). Other parameter values were kept constant at patch size *X*_*H*_ = 50, total number of seeds dispersed per plant *N*_tot_ = 10,000, survival and germination rate *g* = 0.3, seed production cost *c*_1_ = 0.0001, number of generations per fragmentation level *N*_gens_ = 1000, and mutation rate *μ* = 0.001. Arrows indicate the hysteresis zones.

Results from our deterministic and stochastic model confirm that, at least in theory, dispersal capacity can be abruptly lost once habitat patches become too far apart and, likewise, can be gained abruptly again once the distance between such patches is sufficiently decreased (Figures 3 and 4). In most of these cases, there is a hysteresis zone (Figures 3 and 4). At small inter‐patch distances, evolved median dispersal distance increases with growing inter‐patch distance in both models. After this section of increasing dispersal capacity, we observe a gradual decrease in dispersal capacity with further increasing inter‐patch distances in the deterministic model, until a threshold fragmentation level is reached, after which evolved dispersal capacity collapses to a much lower level (a critical transition; Figure 3). In the stochastic model, dispersal capacity is suddenly lost after surpassing a threshold fragmentation level, and no section of gradual loss of dispersal capacity is observed (Figure 4). After this sudden shift from long‐distance to short‐distance dispersal, reversion of habitat fragmentation by decreasing inter‐patch distances does not immediately increase dispersal capacity. Only if inter‐patch distances are sufficiently small, dispersal capacity evolves back to its former state (another critical transition; Figures 3 and 4).

The range of inter‐patch distances in which evolutionary hysteresis of dispersal capacity occurs depends on the used model and parameter values. For the deterministic model, the size of the hysteresis effect increased when the difference in dispersal distances between small and large seeds increased (i.e., when larger seeds dispersed much farther than smaller seeds, the hysteresis effect was strongest; Figure 3). For the stochastic model, the size of the hysteresis effect varied strongly and seemingly more arbitrarily between parameter combinations, though it was mostly large when both small and large seeds dispersed over long distances (i.e., when larger seeds dispersed over long distances relative to the patch size, and small seeds also dispersed over reasonable distances, the hysteresis effect was strongest; Figures 4 and 6). In most model runs, especially those with the stochastic model, dispersal capacity first increases with inter‐patch distances, before it is abruptly lost (Figures 3 and 4).

### 3.2 Sensitivity analysis

**FIGURE 5:**
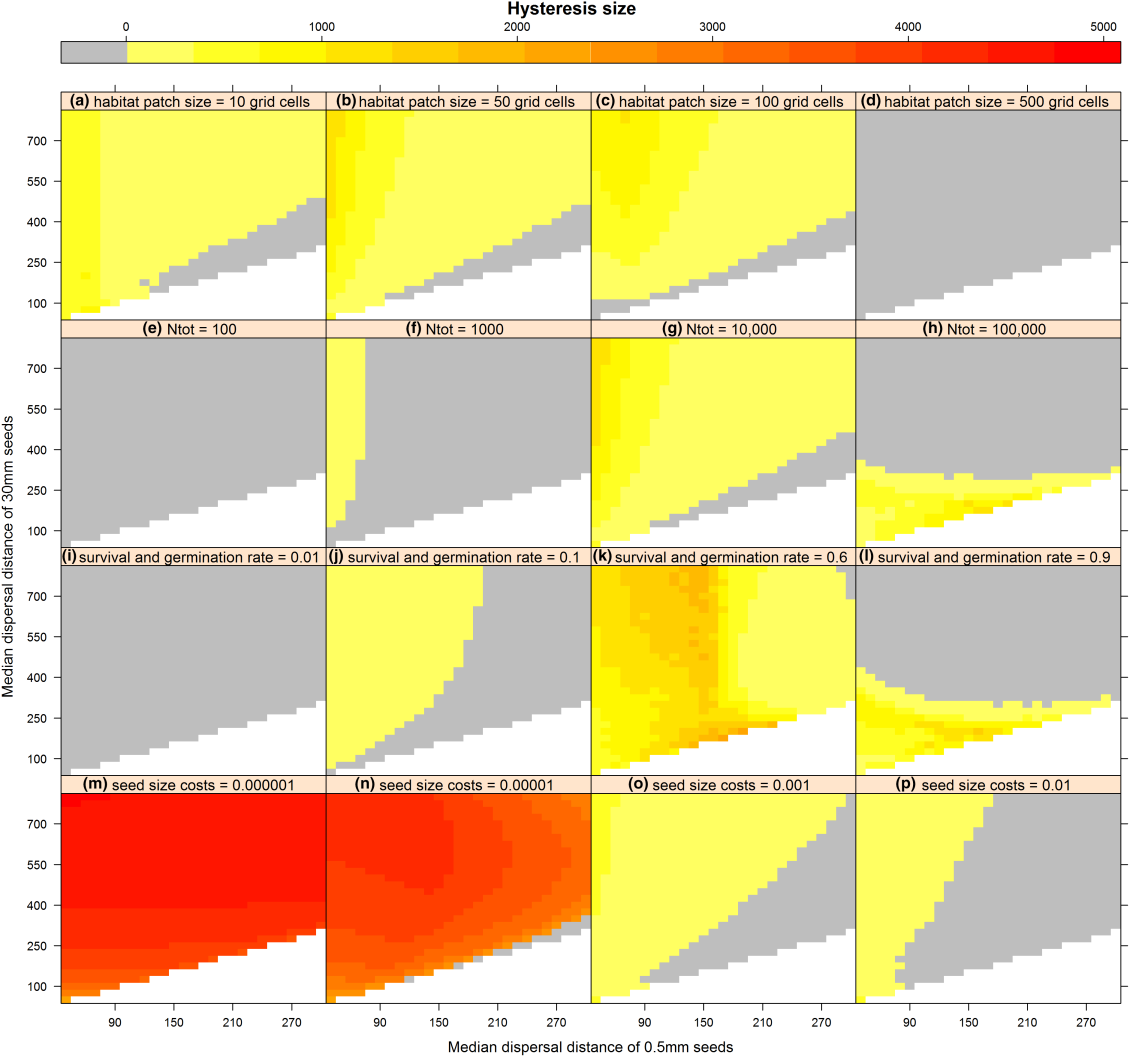
Size of the hysteresis zone (in cells), using the deterministic model, for a range of parameter value combinations, varying the median dispersal distance of 0.5 mm seeds (in cells), the median dispersal distance of 30 mm seeds (in cells), and the habitat patch size (a–d), the total number of seeds (e–h) survival and germination rate (i–l) or the seed size costs (m–p). Gray areas indicate no hysteresis zone; white areas contain no data (*d*_0.5_ ≤ *d*_30_). If not the changing parameter, parameter values used in model runs are *X*_*H*_ = 50, *N*_tot_ = 10,000, *g* = 0.3, and *c*_1_ = 0.0001.

**FIGURE 6:**
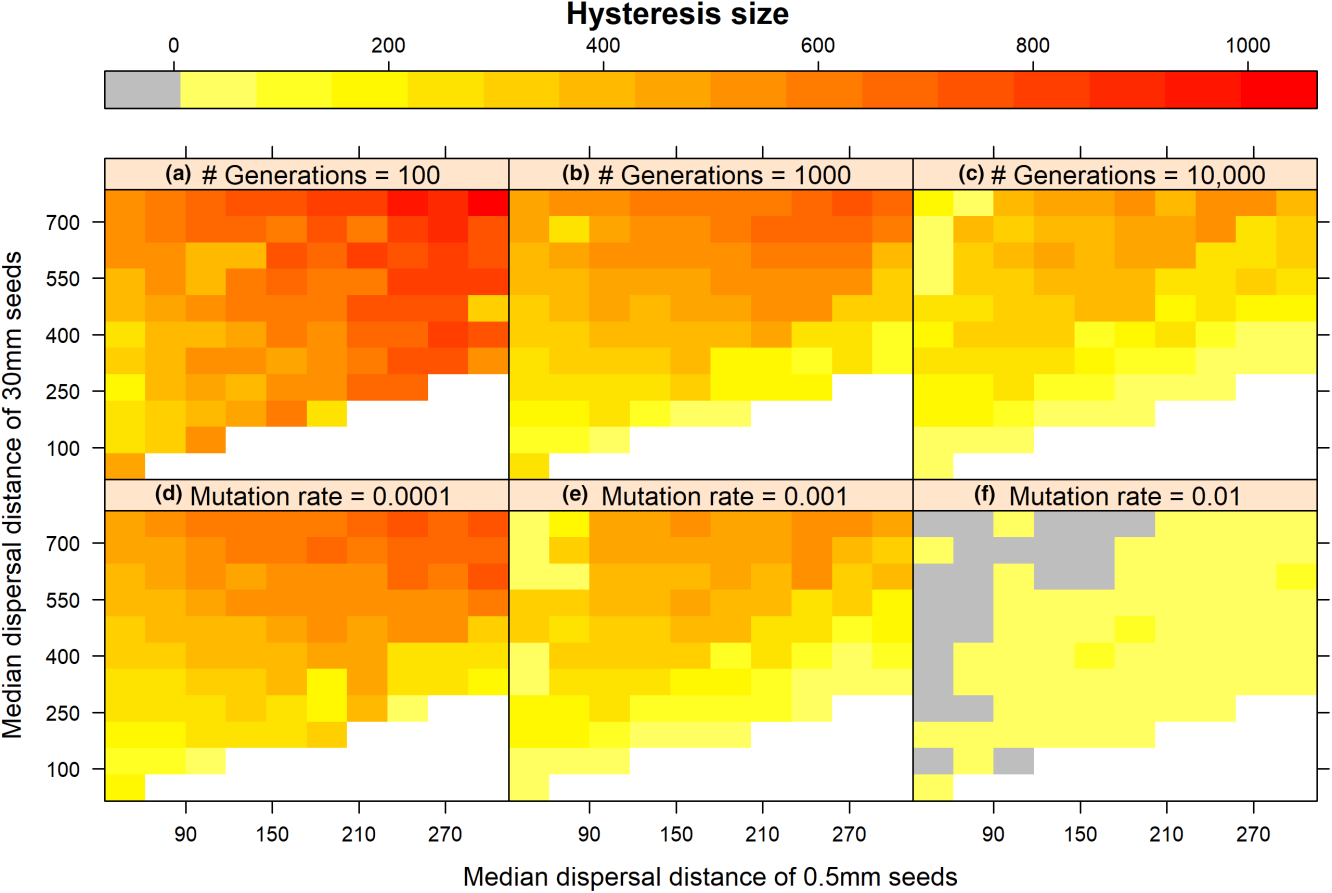
Size of the hysteresis zone (in cells), using the stochastic model, for a range of parameter value combinations, varying the median dispersal distance of 0.5 mm seeds (in cells), the median dispersal distance of 30 mm seeds (in cells), and the number of generations per level of habitat fragmentation (a–c) or the mutation rate (d–f). Gray areas indicate no hysteresis zone; white areas contain no data (*d*_0.5_ ≤ *d*_30_). If not the changing parameter, parameter values used in simulations are *X*_*H*_ = 50, *N*_tot_ = 10,000, *g* = 0.3, *c*_1_ = 0.0001, *N*_gens_ = 10,000, and *μ* = 0.001.

As expected, we did not observe evolutionary hysteresis effects occurring in the entire examined parameter space (Figures 5 and 6). For some parameter combinations, there is a gradual decrease of mean dispersal distance with increasing distances between habitat patches without a sudden drop in dispersal capacity, and a gradual increase back again with decreasing inter‐patch distances. In these cases, the dispersal strategy that evolves does not depend on fragmentation history.

Our sensitivity analyses indicate that, for the deterministic model, no hysteresis was observed when habitat patch sizes were large (*X*_*H*_ = 500 cells; Figure 5d), the total number of seeds small (*N*_tot_ < 1000, Figure 5e), or the survival and germination rate small (*g* = 0.01, Figure 5i). In all other instances, hysteresis was observed for some combinations of median dispersal distances of 0.5‐ and 30‐mm seeds.

When habitat patches were large, long‐distance dispersal remained a good strategy, as few seeds were lost to the uninhabitable matrix between patches. For small‐to‐intermediate habitat patch sizes, we found a parameter space in which small hysteresis in dispersal capacity occurred (Figure 5a–c).

Considering seed number, no hysteresis was found when few seeds were dispersed (Figure 5e). Here, there is little kin competition; hence, short‐distance dispersal is always the best strategy in this scenario. The parameter space in which hysteresis occurred was largest when an intermediate number of seeds was dispersed (Figure 5g).

Similarly, intermediate survival and germination rates also resulted in the largest parameter space with hysteresis occurring (Figure 5k). In case of low survival and germination rates, no hysteresis was observed. Here, kin competition was low as well, resulting in short‐distance dispersal at all examined fragmentation levels.

The largest hysteresis zones were observed when seed size costs were small (*c*_1_ ≤ 0.00001). Here, seed size and thus dispersal capacity is maximized with increasing inter‐patch distances, until a critical threshold is reached, after which the smallest possible seed size—with the shortest dispersal distances—is most efficient, as the least seeds are lost to the uninhabitable matrix between patches.

Simulations with the stochastic model resulted in larger hysteresis zones when time between changes in inter‐patch distances were short (*N*_gens_ = 100; Figure 6a) or mutation rates small (*μ* = 0.001; Figure 6d), for example, when there is insufficient time between environmental changes to adapt to the new situation. With increasing numbers of generations and mutation rates, the size of the inter‐patch distance range in which hysteresis occurs decreases (Figure 6).

### 3.3 When to expect evolutionary hysteresis in dispersal capacity

Based on the results from our deterministic and stochastic models, we can expect evolutionary hysteresis in dispersal capacity caused by changes in habitat fragmentation levels when (i) habitat patches are small to intermediate in size in comparison with a species' maximum number of plants they can accommodate, (ii) the amount of seeds dispersed per plant is quite high (~10,000 seeds), (iii) survival and germination rates are quite high (~0.6), (iv) the investments to increase seed dispersal capacity are relatively low, (v) the number of generations between changes in habitat fragmentation levels is low, and (vi) mutation rates are low. The largest hysteresis zones are expected to occur when dispersal capacity differs substantially between large and small seeds.

## 4 DISCUSSION

Our model suggests that, for species in a very simple system consisting of habitable patches separated by a non‐inhabitable matrix, whose movements are non‐directed and across distances primarily determined by organism traits, increasing habitat fragmentation of relatively small habitat patches may result in an abrupt loss of dispersal ability that seems difficult to reverse. When the distances between habitat patches are small, seed dispersal over long distances evolves. This is in line with general theoretical predictions of dispersal, where long‐distance dispersal strategies are favored in continuous habitats or habitable areas separated only by short distances, due to the avoidance of kin competition (Treep et al., 2021). With increasing distances between habitat areas, seed dispersal capacity increases. By increasing dispersal capacity, subsequent habitat patches may still be reached when inter‐patch distances increase. Yet, when distances between patches become too large, seed dispersal capacity decreases. From this point on, loss of seeds dispersing into the unsuitable matrix is counterbalanced by dispersing more seeds within the parental area. Then, at a certain critical distance between habitable areas, even the slightest additional increase in this distance results in a collapse in seed dispersal capacity: a critical transition from long‐distance to short‐distance dispersal. Interestingly, a similar non‐linear relation between dispersal capacity and landscape fragmentation has also been shown in an earlier theoretical study, which demonstrated that landscape connectivity may be abruptly lost if model organisms gradually decrease their dispersal capacity (Keitt et al., 1997).

A crucial result from our models is that the distance between habitat patches may need to be greatly reduced to effectively restore movement between habitat fragments. In other words, the connectivity between patches needs to be restored to a relatively high level in order to allow an evolutionary trajectory toward long‐distance dispersal. As mutations result in small changes in a trait such as seed size, evolution of seed size may not occur through fitness valleys, resulting in a hysteresis zone in seed size. When the distance between habitat patches is decreased but the system remains within the hysteresis zone, short‐distance dispersal strategies will remain the evolutionary attractor, as small increases in seed size, and thereby in dispersal distance, result in lower relative fitness (Figure S1). Once the distance between habitat patches has been effectively reduced, the valley in the fitness landscape is no longer present, and small increases in dispersal capacity result in increases in fitness. This process leads to selection of seeds that can disperse over longer distances, effectively reconnecting habitat across the landscape.

Hysteresis in dispersal evolution may explain lack of restoration success due to failure of species to recolonize restored areas in heavily fragmented landscapes where remnant source populations still survive but have been isolated for a relatively long period of time. As biodiversity is under threat throughout the world and populations are being increasingly fragmented (IPBES, 2019), it is of critical importance to restore connectivity between habitat patches so that species can overcome possible hysteresis effects and disperse again between habitat fragments. Our study shows that these connectivity levels may need to be much higher than minimal connectivity levels maintaining dispersal in reference populations in relatively pristine landscapes.

Critical transitions from long‐ to short‐distance dispersal in natural populations have not yet been observed, mainly due to the suddenness of the shift and the absence of empirical and experimental studies investigating the effect of increasing habitat fragmentation on dispersal evolution over time. For plant species, Cheptou et al. (2008) detected a substantial difference in dispersal capacity between *Crepis sancta* populations in a city and a less fragmented rural area, where short‐range dispersal seemed to have evolved in the more fragmented landscape of the city (Cheptou et al., 2008). This study also illustrated one of the mechanisms by which rapid loss of dispersal may evolve: a shift in the ratio between two distinct types of propagules, one with appendages to enhance dispersal and one without, on the same plant. Many plant species (>50 genera) within the Asteraceae family exhibit such a seed dimorphism (Imbert, 2002), suggesting that rapid evolution of dispersal may be common in populations of this large group of generally wind‐dispersed plant species and that species from this family might be a suitable model system to record the first experimental evidence for critical transitions in dispersal.

Though both our deterministic and stochastic models provide a proof‐of‐principle of potential evolutionary hysteresis in dispersal capacity caused by changes in habitat fragmentation, their results are somewhat different from one another. As expected, dispersal capacity changes more gradually in the deterministic model, as stochasticity is omitted here. Due to its nature, the stochastic model provides more variable results, and different outcomes can be reached while using the same parameter combinations. An interesting result is that, in the stochastic model, long‐distance dispersal did often not re‐evolve when decreasing inter‐patch distances. This may be caused by the extreme competition for space that occurs in the model; most, if not all, habitat patches were occupied during every generation. If we would introduce a small probability of patch turnover, colonization of new habitat would likely result in an easier evolutionary shift back to long‐distance dispersal (Treep et al., 2021).

We selected a well‐studied model system that is characterized by unidirectional dispersal with an exponential dispersal kernel. The simplified setup that we chose for our first theoretical proof‐of‐principle of evolutionary hysteresis in dispersal capacity can be improved and extended upon in future studies. For instance, we created a one‐dimensional environment, while most dispersal occurs in two‐dimensional habitats. Expanding the model used in Treep et al. (2021) may show that the critical transitions we observe in our simulated 1‐D environments can also occur in 2‐D environments. Also, while our simulated habitat patches were of equal quality throughout the modeled space, habitat quality is highly diverse in real landscapes. In fact, reduction in habitat quality rather than a complete loss of habitat is generally underlying habitat degradation and fragmentation. In future studies, one might include variation in habitat quality when modeling eco‐evolutionary dynamics of seed dispersal (Liao, Li, Hiebeler, Iwasa, et al., 2013; Mortelliti et al., 2010). Also, we placed habitat patches equidistantly from another. Yet, clustering of habitat patches is highly likely to result in different ecological and evolutionary outcomes (Hiebeler, 2000; Liao, Li, Hiebeler, Iwasa, et al., 2013). In such heterogeneous environments, multiple dispersal strategies can coexist, and community composition can vary substantially between locations (Cote et al., 2017; Hiebeler, 2007; Liao, Li, Hiebeler, El‐Bana, et al., 2013; Liao, Li, Hiebeler, Iwasa, et al., 2013). An extension of our model may show us the effects of spatial heterogeneity on dispersal evolution.

Our model is based on an exponential dispersal kernel, which is typical for seeds dispersed by water. Many plant species, across a range of dispersal mechanisms, have even more leptokurtic dispersal distributions that are characterized by fat tails (Bullock et al., 2017). However, in plant species that are dispersed by vertebrates, the navigation capacity of the vector may play an important role in seed dispersal, especially in fragmented landscapes where large vertebrates are able to maintain connections between otherwise isolated patches (Kleyheeg et al., 2017). Such species are likely to show a very slow evolutionary trait trajectory toward limited or restored movement and may lack a hysteresis in response to defragmentation. Also, including habitat dynamics (i.e., turnover of habitable areas) is likely to significantly affect the outcomes of our model. Slow habitat dynamics (i.e., patch life span much exceeding organism life span) will result in evolution of short‐distance and long‐distance dispersal in much the same way as in permanent habitable areas (Treep et al., 2021). However, long‐distance dispersal is essential for survival in highly dynamic habitats (Treep et al., 2021). If habitat fragmentation in such environments exceeds a certain threshold, evidence suggests that population extinction rather than loss of dispersal is the likely outcome (Fountain et al., 2016; Treep et al., 2021). An empirical study on extinct Glanville fritillary butterfly populations shows that dispersal capacity was enhanced instead of inhibited in response to increased habitat fragmentation, before regional collapse of dispersal capacity and extinction of the species in the most disturbed regions occurred (Fountain et al., 2016). Some of our results of the stochastic model show the same patterns as observed in this study. In this species, the sudden collapse of dispersal capacity and extinction of the population coincided, which is in line with the result of model simulations with high habitat dynamics (Treep et al., 2021). Based on the exploratory work presented here, we suggest that new lines of research should include unraveling the relations between habitat fragmentation and dispersal limitation in (i) organisms with directed dispersal and (ii) in highly dynamic habitats, besides the (iii) empirical testing of our hypotheses in existing and experimental populations. It is of urgent importance to extend this research and find further validation and empirical support to prove that our proposed mechanism is responsible for the observed discrepancies between habitat restoration and actual colonization, thereby providing new conservation strategies that are needed to overcome the ecological and evolutionary consequences of habitat fragmentation.

## AUTHOR CONTRIBUTIONS

**Monique de Jager:** Conceptualization (lead); formal analysis (lead); methodology (lead); writing – original draft (lead). **Merel Soons:** Conceptualization (supporting); supervision (lead); writing – review and editing (lead).

## ACKNOWLEDGMENTS

We thank George Kowalchuk, Yann Hautier, and Bart Nolet for constructive feedback.

## Supporting information


Appendix S1

Appendix S2

Figure S1

## Data Availability

Simulated datasets: Dryad doi: 10.5061/dryad.rfj6q57f9. C++ scripts: Appendix S1 (deterministic model) and Appendix S2 (stochastic model).

## APPENDIX 1

In this paper, we parameterized the dispersal kernel using the median dispersal distances of the smallest and largest seeds considered in our model, as these parameters are ecologically meaningful and more intuitive to interpret than the parameters *α* and *β*. To quantify variation in parameters *α* and *β* in terms of median dispersal distances *d*_0.5_ and *d*_30_, we here provide an explanation of how *α* and *β* can be derived from *d*_0.5_ and *d*_30._ The fraction of seeds remaining in the water column after dispersing a distance *d* (in cells) follows an exponential decay function:

(A1)
$$F_{d}=\lambda^{d},$$

where *λ* is the decay exponent (de Jager et al., 2019). Following de Jager et al. (2019), we assume that *λ* is a sigmoidal function of seed size (*S*):

(A2)
$$\lambda_{S}=1-\frac{1}{1+e^{\alpha +\beta \left(S-0.5\right)}}$$

where *α* and *β* are the model parameters that define the slope and shape of the sigmoid curve (de Jager et al., 2019). The 0.5 in equation 2 represents the minimum seed size (0.5 mm), for which *β(S‐0.5)* = 0. As *α* and *β* are not ecologically meaningful and therefore not easily interpreted, we hereafter parameterized the dispersal kernel using the median dispersal distances (*F*_*d*_ = 0.5) of the smallest and largest seeds considered in our model (0.5 and 30 mm, respectively): *d*_0.5_ and *d*_30_ at *F*_*d*_ = 0.5. When seed size *S* = 0.5 mm, equation A2 results in $\lambda_{0.5}=1-\frac{1}{1+e^{\alpha +\beta \left(0.5-0.5\right)}}$, which can be rewritten as

(A3)
$$\lambda_{0.5}=1-\frac{1}{1+e^{\alpha }}$$

Reorganizing this equation gives

$$\frac{1}{1+e^{\alpha }}=1-\lambda_{0.5},$$

$$1+e^{\alpha }=\frac{1}{1-\lambda_{0.5}},$$

$e^{\alpha }=\frac{1}{1-\lambda_{0.5}}-1$, and finally

(A4)
$$\alpha =\ln\left(\frac{1}{1-\lambda_{0.5}}-1\right).$$

As the median dispersal distance occurs at *F*_*d*_ = 0.5, we can solve for *λ* ^0.5^:

$$F_{d_{0.5}}={\lambda_{0.5}}^{d_{0.5}},$$

$$0.5={\lambda_{0.5}}^{d_{0.5}},$$

$$d_{0.5}=\log_{\lambda_{0.5}}0.5,$$

$$d_{0.5}=\frac{\ln\left(0.5\right)}{\ln\left(\lambda_{0.5}\right)},$$

$$\ln\left(\lambda_{0.5}\right)=\frac{\ln\left(0.5\right)}{d_{0.5}},$$

(A5)
$$\lambda_{0.5}=e^{\ln\left(0.5\right)/d_{0.5}}$$

When seed size *S* = 30 mm, equation A2 results in

(A6)
$$\lambda_{30}=1-\frac{1}{1+e^{\alpha +\beta \left(30-0.5\right)}},$$

Reorganizing this equation gives

$$\frac{1}{1+e^{\alpha +29.5\beta }}=1-\lambda_{30},$$

$$1+e^{\alpha +29.5\beta }=\frac{1}{1-\lambda_{30}},$$

$$e^{\alpha +29.5\beta }=\frac{1}{1-\lambda_{30}}-1,$$

$$\alpha +29.5\beta =\ln\left(\frac{1}{1-\lambda_{30}}-1\right),$$

$$29.5\beta =\ln\left(\frac{1}{1-\lambda_{30}}-1\right)-\alpha ,$$

(A7)
β=129.5∙ln11−λ30−1−α,

As the median dispersal distance occurs at *F*_*d*_ = 0.5, we can solve for *λ*_30_:

$$F_{d_{30}}={\lambda_{30}}^{d_{30}},$$

$$0.5={\lambda_{30}}^{d_{30}},$$

$$d_{30}=\log_{\lambda_{30}}0.5,$$

$$d_{30}=\frac{\ln\left(0.5\right)}{\ln\left(\lambda_{30}\right)},$$

$$\ln\left(\lambda_{30}\right)=\frac{\ln\left(0.5\right)}{d_{30}},$$

(A8)
$$\lambda_{30}=e^{\ln\left(0.5\right)/d_{30}}.$$

This allows us to establish a link between the more intuitive and ecologically relevant parameters *d*_0.5_ and *d*_30_ and the parameters *α* and *β* (used in de Jager et al., 2019).
