# Supplementary material for: Critical transitions and evolutionary hysteresis in movement: Habitat fragmentation can cause abrupt shifts in dispersal that are difficult to revert
Source: Ecol Evol. 2023 May 29;13(5):e10147. doi: 10.1002/ece3.10147 (PMC10227176; doi:10.1002/ece3.10147)
Supplement: Supplementary file 1 — Appendix S1 [file ECE3-13-e10147-s001.pdf]

Supplement A: C++ code for deterministic model

'Critical transitions and evolutionary hysteresis in movement: Habitat fragmentation can cause abrupt shifts in dispersal that are difficult to revert' by Monique de Jager & Merel Soons

```
#include <iostream>
#include <fstream>
#include <string>
#include <algorithm>
#include <vector>
#include <ctime>
#include <cstdlib>
#include <random>
#include <math.h>

using namespace std;

// Functions used in the model (codes are found below the main program in alphabetical order):
double calcW(double, int);
void terminateProgram();
void writeData1(double, double, int, double);
void writeData2(double, double, int, double, double);

// global parameters
double a, b, d = 0.9, c = 0.0001;
int Ntot = 10000, Xmax = 50000, patchSize = 50, modelType = 0;
int simNumber = 0;
double Dist0 = 40;
double Dist30 = 750;

// modelType = 0 is the model used to follow evolution of seed size, given the seed size at a slightly smaller or
larger inter-patch distance
// modelType = 1 is the model used to create the input for figure3b, c, and d

// Dist0 is the distance dispersed by the 50-percentile of 0.5mm seeds
// Dist30 is the distance dispersed by the 50-percentile of 30mm seeds

// main program:
int main() {
    if (modelType == 0) {

        double L0 = exp(log(0.5) / Dist0);
        a = log(1 / (1 - L0) - 1);
        double L30 = exp(log(0.5) / Dist30);
        b = (log(1 / (1 - L30) - 1) - a) / 29.5;

        bool first = true;
        double S = 30;
        double S1[10000], S2[10000];
        for (int gapSize = 0; gapSize < 10000; ++gapSize) {
            double Wres = calcW(S, gapSize);
            double WmutPlus = calcW((S + 0.01), gapSize);
            double WmutMin = calcW((S - 0.01), gapSize);
```

```
54
55                  while ((WmutPlus > Wres) && (S <= 30)) {
56                          S = S + 0.01;
57                          Wres = calcW(S, gapSize);
58                          WmutPlus = calcW((S + 0.01), gapSize);
59                  }
60
61                  while ((WmutMin > Wres) && (S > 0.5)) {
62                          //cout << WmutMin << " > " << Wres << endl;
63                          S = S - 0.01;
64                          Wres = calcW(S, gapSize);
65                          WmutMin = calcW((S - 0.01), gapSize);
66                  }
67
68                  S1[gapSize] = S;
69                  cout << S << " ; " << gapSize << endl;
70                  writeData1(Dist0, Dist30, gapSize, S);
71          }
72
73          for (int gapSize = 9999; gapSize >= 0; --gapSize) {
74                  double Wres = calcW(S, gapSize);
75                  double WmutPlus = calcW((S + 0.01), gapSize);
76                  double WmutMin = calcW((S - 0.01), gapSize);
77
78                  while ((WmutPlus > Wres) && (S <= 30)) {
79                          S = S + 0.01;
80                          Wres = calcW(S, gapSize);
81                          WmutPlus = calcW((S + 0.01), gapSize);
82                  }
83
84                  while ((WmutMin > Wres) && (S > 0.5)) {
85                          //cout << WmutMin << " > " << Wres << endl;
86                          S = S - 0.01;
87                          Wres = calcW(S, gapSize);
88                          WmutMin = calcW((S - 0.01), gapSize);
89                  }
90
91                  S2[gapSize] = S;
92
93                  writeData1(Dist0, Dist30, gapSize, S);
94                  cout << S << " ; " << gapSize << endl;
95          }
96
97          double dS[10000];
98          int hysteresis = 0;
99          for (int i = 0; i < 10000; ++i) {
100                 dS[i] = S1[i] - S2[i];
101                 if (dS[i] != 0)
102                         ++hysteresis;
103         }
104         cout << Dist0 << "        " << Dist30 << " " << hysteresis << endl;
105
106 }
```

```
107
108     if (modelType == 1) {
109         // generate the data used to make figure 3b, c, and d:
110         int gapSize = 500;
111         double Dist0 = 40;
112         double Dist30 = 750;
113         double L0 = exp(log(0.5) / Dist0);
114         a = log(1 / (1 - L0) - 1);
115         double L30 = exp(log(0.5) / Dist30);
116         b = (log(1 / (1 - L30) - 1) - a) / 29.5;
117         for (int gapSize = 500; gapSize < 1501; gapSize += 500) {
118             for (double S = 0.5; S < 30.1; S += 0.1) {
119                 double W = calcW(S, gapSize);
120                 writeData2(Dist0, Dist30, gapSize, S, W);
121             }
122         }
123     }
124
125     // terminate program:
126     terminateProgram();
127 }
128
129 double calcW(double S, int gapSize) {
130     // given seed size S and gap size 'gapSize', what is the average number of patches occupied
131     // by seeds of one individual?
132     double lambda = 1 - 1 / (1 + exp(a + b * (S - 0.5)));
133     double C = Ntot / (pow(lambda, Xmax) - pow(lambda, 0));
134     double totP = 0;
135
136     for (int i = 1; i <= Xmax; ++i) {
137       if (i % (patchSize + gapSize) < patchSize) {
138             double Nx = C * (pow(lambda, (i + 1)) - pow(lambda, i));
139             double Px = 1 - pow(d, (Nx * (1 - c * 3.14 / 6 * pow((S - 0.5), 3))));
140             totP += Px;
141       }
142     }
143     //cout << totP << " " << S << " " << gapSize << endl;
144     return(totP);
145 }
146
147
148 void terminateProgram() {
149         cout << "Press any character and <ENTER> to continue" << endl;
150         char chAnyChar;
151         cin >> chAnyChar;
152         return;
153 }
154
155 void writeData1(double Dist0, double Dist50, int gapsize, double S) {
156         ofstream outFile;
157         string filename = "dataSeedSizePerGapSize.txt";
158         outFile.open(filename, ios::app);
159         outFile << Dist0 << "; " << Dist50 << "; " << gapsize << "; " << S << endl;
```

```
160         outFile.close();
161     }
162
163     void writeData2(double Dist0, double Dist30, int gapSize, double S, double W) {
164         ofstream outFile;
165         string filename = "dataFitnessPerSeedSizePerGapSize.txt";
166         outFile.open(filename, ios::app);
167         outFile << Dist0 << "; " << Dist30 << "; " << gapSize << "; " << S << "; " << W << endl;
168         outFile.close();
169     }
```
