# Supplementary material for: Critical transitions and evolutionary hysteresis in movement: Habitat fragmentation can cause abrupt shifts in dispersal that are difficult to revert
Source: Ecol Evol. 2023 May 29;13(5):e10147. doi: 10.1002/ece3.10147 (PMC10227176; doi:10.1002/ece3.10147)
Supplement: Supplementary file 2 — Appendix S2 [file ECE3-13-e10147-s002.pdf]

Supplement B: C++ code for stochastic model

'Critical transitions and evolutionary hysteresis in movement: Habitat fragmentation can cause abrupt shifts in dispersal that are difficult to revert' by Monique de Jager & Merel Soons

```
#include <iostream>
#include <fstream>
#include <string>
#include <algorithm>
#include <vector>
#include <ctime>
#include <cstdlib>
#include <random>
#include <math.h>

using namespace std;

// Functions used in the model (codes are found below the main program in alphabetical order):
void calcNseeds();                  // calculate the number of seeds that will be dispersed per plant
void createLandscape();             // create the landscape
void initializeSeedSize();          // initializes the first simulation's seed size
void mutate();                      // let seed sizes mutate with mutation rate mutRate
void runSimulation();               // runs the actual simulation
void writeOutput(int gen);          // write seed size info to a file
void terminateProgram();

// global parameters
const int patchSize = 50;           // size of habitable patches (in number of locations)
const int nPatches = 10;            // number of patches in the modelled environment (continuous
                                    // boundaries, seeds disperse from end to beginning of stream when
                                    // the maximum distance is exceeded)
int nGenerations = 10000;           // number of generations to simulate
int locationX[patchSize * nPatches];          // x-coordinate of each location
double locationS[patchSize * nPatches];       // seed size of plant growing at each location
double locationN[patchSize * nPatches];       // number of seeds that the plant growing at this location will
                                              // disperse
double locationSnewGen[patchSize * nPatches];     // seed size of new generation plant (= succesful seed)
                                                  // growing at each location
int totalN;                         // total number of seeds available for dispersal
int simNumber = 0;                  // to get the right parameter values from the parameter value input file

double d = 0.9;                     // death rate of seeds at habitable patches
double c = 0.0001;                  // cost of seed size volume
double a, b;
int nSeeds = 1000;                  // number of seeds without considering the cost c
int gapSize = 1;                    // size of uninhabitable area between two patches
double maxDist;
double iniS = 20;                   // initial seed size; should depend on the last simulation
double mutRate = 0.001;             // mutation rate, probability that a seed mutates it's offsprings' seed size by
                                    // mutSize mm
double mutSize = 1;

double Dist0 = 40;                  // Dist0 is the distance dispersed by the 50-percentile of 0.5mm seeds
```

```
54  double Dist30 = 750;                    // Dist30 is the distance dispersed by the 50-percentile of 30mm seeds
55
56  // main program:
57  int main()
58  {
59          double L0 = exp(log(0.5) / Dist0);
60          a = log(1 / (1 - L0) - 1);
61          double L30 = exp(log(0.5) / Dist30);
62          b = (log(1 / (1 - L30) - 1) - a) / 29.5;
63
64          srand(time(NULL));
65          initializeSeedSize();
66          for (gapSize = 5; gapSize <1001; gapSize = gapSize + 5) {
67                  createLandscape();
68                  runSimulation();
69          }
70          for (gapSize = 1000; gapSize > 4; gapSize = gapSize - 5) {
71                  createLandscape();
72                  runSimulation();
73          }
74
75          terminateProgram();
76  }
77
78  void calcNseeds() {
79          // calculate the number of seeds that will be dispersed per plant
80          totalN = 0;
81          for (int i = 0; i < (nPatches * patchSize); ++i) {
82                  if (locationS[i] > 0) {
83                          double nS = nSeeds * (1.0 - c * 3.14 / 6.0 * pow((locationS[i] - 0.5), 3));
84                          locationN[i] = nS * (1 - d);  // take only the surviving seeds in account (doesn't matter
85                                                        // if we do this now already or after dispersal)
86                  }
87                  else { locationN[i] = 0; }
88                  if (locationN[i] > 0) { totalN = totalN + floor(locationN[i]); }
89          }
90  }
91
92  void createLandscape()
93  {
94          // create the landscape
95          int locID = 0;
96          for (int i = 1; i <= nPatches; ++i) {
97                  for (int j = 0; j < patchSize; ++j) {
98                          int x = i * (gapSize + patchSize) - patchSize + j;
99                          locationX[locID] = x;
100                         ++locID;
101                 }
102         }
103         maxDist = ((double)gapSize + (double)patchSize) * (double)nPatches;
104 }
105
106 void initializeSeedSize() {
```

```
107             // initializes the first simulation's seed size
108             for (int i = 0; i < (nPatches * patchSize); ++i) { locationS[i] = iniS; }
109     }
110
111     void mutate() {
112             int nPlus = 0, nMin = 0, nPlants = 0;
113             for (int i = 0; i < (nPatches * patchSize); ++i) {
114                     // seeds produce next generation:
115                     locationS[i] = locationSnewGen[i];
116                     if (locationS[i] > 0) {
117                             ++nPlants;
118
119                             // let their offsprings' seed sizes mutate with mutation rate mutRate:
120                             if (((rand() % 100000) / 100000.0) <= mutRate) {
121                                     // a mutation occurs
122                                     if ((rand() % 2) == 0) {
123                                             // it is a + mutation
124                                             ++nPlus;
125                                             locationS[i] = locationS[i] + mutSize;
126                                     }
127                                     else {
128                                             // it is a - mutation
129                                             ++nMin;
130                                             if (locationS[i] > mutSize) { locationS[i] = locationS[i] - mutSize; }
131                                     }
132                             }
133                     }
134             }
135     }
136
137     void runSimulation() {
138             // run the actual simulation
139             for (int gen = 0; gen < nGenerations; ++gen) {
140                     // calculate number of seeds to disperse per plant (already decrease this number by
141                     // accounting for the death rate):
142                     calcNseeds();
143
144                     // disperse seeds in random order (start without new generation)
145                     for (int i = 0; i < (nPatches * patchSize); ++i) { locationSnewGen[i] = 0; }
146
147                     bool disperse = true;        // whether we should keep on dispersing seeds
148                     if (totalN == 0) { disperse = false; }
149
150                     int locAvailable = (nPatches * patchSize);
151                     while (disperse) {
152                             int x = rand() % (nPatches * patchSize); // randomly selected plant at patch x
153                             while (floor(locationN[x]) <= 0) {
154                                     // select another plant if all seeds have been dispersed already for this plant
155                                     ++x;
156                                     if (x >= (nPatches * patchSize)) { x = 0; }
157                             }
158
159                             // draw dispersal distance from frequency distribution:
```

```
160                                 double lambda = 1 - 1 / (1 + exp(a + b * (locationS[x] - 0.5)));
161                                 double Fd = (rand() % 10000) / 10000.0;
162                                 if (Fd < 0.0000001) { Fd = 0.0000001; }
163                                 double dist = log(Fd) / log(lambda);
164                                 int direction = 1;
165                                 if ((rand() % 2) == 1) { direction = -1; }
166                                 int newloc = locationX[x] + round(dist * direction);
167
168                                 while (newloc > maxDist) { newloc = newloc - maxDist; }
169                                 while (newloc < 0) { newloc = maxDist + newloc; }
170
171                                 // is this an available habitable location?
172                                 double a1 = (double)newloc / ((double)gapSize + (double)patchSize);
173                                 double a2 = (a1 - floor(a1)) * ((double)gapSize + (double)patchSize) –
174                                 (double)gapSize;
175
176                                 a2 = round(a2 * 100.0) / 100.0;
177                                 if ((a2 < 0) | (a2 > ((double)patchSize - 0.99))) {
178                                         // location is unsuitable
179                                 }
180                                 else {
181                                         // location is suitable habitat; is it available?
182                                         int j = floor(a1) * patchSize + round(a2);
183                                         if (locationSnewGen[j] == 0) {
184                                                 // location is available
185                                                 locationSnewGen[j] = locationS[x];
186                                                 --locAvailable;
187                                         }
188                                 }
189                                 locationN[x] = locationN[x] - 1;
190                                 --totalN;
191
192                                 if (totalN == 0) { disperse = false; }
193                                 if (locAvailable == 0) { disperse = false; }
194                         }
195
196                         // mutate the new plants' seed size:
197                         mutate();
198
199                         // write seed size info to a file:
200                         writeOutput(gen);
201                 }
202         }
203
204         void terminateProgram()
205         {
206                 cout << "Press any character and <ENTER> to continue" << endl;
207                 char chAnyChar;
208                 cin >> chAnyChar;
209                 return;
210         }
211
212         void writeOutput(int gen){
```

```
213             // write seed size info to a file every 100 generations:
214
215             if ((round(((double)gen + 1) / 100.0) - (((double)gen + 1) / 100.0)) == 0) {
216                     // what seed sizes are occurring?
217                     int nCounted = 0;
218                     bool counted[nPatches * patchSize];
219                     int j = 0;
220
221                     for (int i = j; i < (nPatches * patchSize); ++i) { counted[i] = false; }
222
223                     while (nCounted < (nPatches * patchSize)) {
224                             while (counted[j]) { ++j; }
225
226                             double outSeedSize = locationS[j];
227                             int outNumber = 0;
228
229                             for (int i = j; i < (nPatches * patchSize); ++i) {
230                                     if (locationS[i] == outSeedSize) {
231                                             ++outNumber;
232                                             ++nCounted;
233                                             counted[i] = true;
234                                     }
235                             }
236                             ++j;
237
238                             if ((gen + 1) == nGenerations) {
239                                     // write the seed sizes to a file:
240                                     ofstream outfile;
241                                     string filename = "./2.
242     Output/includingStochasticity_SeedSizeDistributionsPerGapSize_" + to_string(inputNr) + "_" +
243     to_string(simNumber) + "_2.txt";
244                                     outfile.open(filename, std::ios_base::app);
245
246                                     outfile << patchSize << " ; " << d << " ; " << c << " ; " << nSeeds << " ; " <<
247     nGenerations << " ; " << mutRate << " ; " << mutSize << " ; " << Dist0 << " ; " << Dist30 << " ; " << gapSize << " ; "
248     << outNumber << " ; " << outSeedSize << endl;
249
250                                     outfile.close();
251                             }
252                     }
253             }
254     }
```
