# Supplementary material for: Critical transitions and evolutionary hysteresis in movement: Habitat fragmentation can cause abrupt shifts in dispersal that are difficult to revert
Source: Ecol Evol. 2023 May 29;13(5):e10147. doi: 10.1002/ece3.10147 (PMC10227176; doi:10.1002/ece3.10147)
Supplement: Supplementary file 3 — Figure S1 [file ECE3-13-e10147-s003.pdf]

Supplementary Figure 1

'Critical transitions and evolutionary hysteresis in movement: Habitat fragmentation can cause abrupt shifts in dispersal that are difficult to revert' by Monique de Jager & Merel Soons

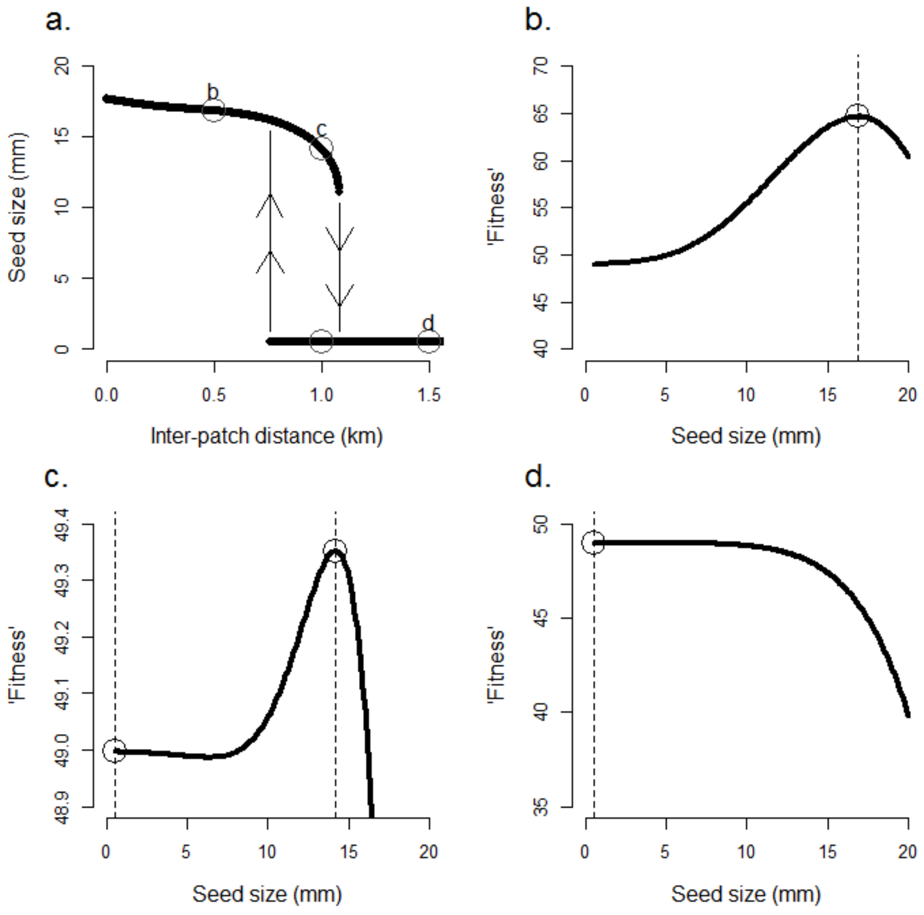

Supplementary Figure 1: *Evolution of evolutionarily stable dispersal strategy in relation to habitat fragmentation, with evolutionary stable strategies per distance between patches in (**a**). Optimal seed sizes based on number of occupied grid cells are illustrated for three different fragmentation levels, represented by distances between habitat patches of 500 (**b**), 1,000 (**c**), and 1,500 (**d**) grid cells (0.5, 1.0, and 1.5 km, respectively). Open circles in (**a**) indicate how these are translated to the hysteresis graph, where two different dispersal strategies can occur when a fitness valley is present in the relation between number of occupied grid cells and seed size (**c**). Parameter values used in these model runs are* X_H *= 50,* N_{tot} *= 10,000, g = 0.1,* c_1 *= 0.0001,* $d_{0.5}$ *= 40 and* d_{30} *= 750.*
